# Establishment of an Inferred Reference Range for Blood Ammonia in Dogs and Cats Using a Point-of-Care Assay

**DOI:** 10.3390/vetsci12060596

**Published:** 2025-06-17

**Authors:** Giulia Specchia, Emily Hannah Doran Seidel, Charlotte Dye

**Affiliations:** 1 Pride Veterinary Referrals, IVC Evidensia, Riverside Rd, Derby DE24 8HX, UK; 2 Creature Comforts Chiswick, 376 Chiswick High Rd., Chiswick, London W4 5TF, UK

**Keywords:** ammonia, point-of-care, hepatic encephalopathy, reference range, internal medicine, emergency

## Abstract

Point-of-care (POC) tests for blood ammonia (BA) measurement have not been well evaluated in veterinary species. This cross-sectional study sought to establish an inferred reference interval for BA using a POC analyser in dogs and cats. Blood ammonia was measured in 175 dogs and 63 cats for which relevant clinical history and laboratory data was available. Reference values were inferred based on comparisons between patients with and without disease pathologies reported to cause BA elevation. Descriptive statistics, Pearson Chi^2^, and Mann–Whitney U testing were used to assess for associations between clinical parameters and BA concentration. Seventy-one percent (124/175) of dogs and forty-six percent (29/63) of cats had undetectable BA. Following the exclusion of dogs with potential causes of hyperammonaemia, all remaining dogs had BA < 30 µg/dL. With one exception, all dogs with BA > 30 µg/dL had liver disease. All dogs with a clinical suspicion of hepatic encephalopathy (HE) had BA > 40 µg/dL. Following the exclusion of cats with potential causes of hyperammonaemia, all remaining cats had BA < 25 µg/dL. Only 50% of cats with BA > 25 µg/dL had liver disease. All cats with a clinical suspicion of HE had BA > 30 µg/dL. Based on this study population, BA < 30 µg/dL and <25 µg/dL should be considered normal in dogs and cats, respectively. Additionally, dogs with BA > 30 µg/dL are likely to have liver disease, while cats with BA > 25 µg/dL appear to exhibit a wider variety of disease pathologies.

## Simple Summary

Measuring blood ammonia is important in the context of some diseases, particularly when they affect the liver; however, the existing methods of measurement are complicated and subject to artefact. A simple and rapid test has been developed and previously used in dogs and cats, but reference values are not yet available. We used this test in groups of dogs and cats with many different diseases, seeking to differentiate those with normal and abnormal values. Where present, we also attempted to document the cause of ammonia elevation. Inferred reference values were developed for both species, and we found that, while most dogs with high ammonia had liver disease, cats with high ammonia exhibited a much wider variety of causes.

## 1. Introduction

Ammonia (NH_3_) is a lipophilic and highly liposoluble substance produced primarily as a consequence of protein metabolism by the gastrointestinal flora [1]. It is readily absorbed through the gastrointestinal wall and transported to the liver through the portal circulation in the form of ammonium ions (NH_4+_) [2]. In periportal hepatocytes, the urea cycle is responsible for detoxification of ammonium ions to urea [3], which is transferred to the systemic circulation and excreted primarily through the kidneys [2]. Other organs take part in the metabolism of ammonia via the glutaminase–glutamine synthetase pathway; these include the kidneys [4], muscles [5], and brain [6].

In human medicine, hyperammonaemia constitutes a significant health concern. Most notably, the prevalence of overt (30–40%) and covert (20–80%) hepatic encephalopathy (HE) is high in patients with hepatic cirrhosis, both of which have major prognostic and economic implications [7]. While hyperammonaemia is classically associated with hepatic failure [8] or with conditions that prevent delivery of nitrogen to the liver (e.g., portosystemic bypass and patent ductus venosus), other notable causes include congenital defects, metabolic diseases, energy-deficient states and blood transfusion. These can be categorised into those affecting nitrogen metabolism and excretion, and those with metabolites or toxins that inhibit urea-cycle function or prevent adequate energy for its normal function. Inherited errors of metabolism include defects in urea-cycle enzymes [9], specific organic acidaemias (e.g., methylmalonic acidaemia and propionic acidaemia; [10]), fatty acid oxidation defects [11], mitochondrial disease [12], and carnitine transporter defects [13]. Acquired conditions can increase ammonia production by stimulating protein catabolism (e.g., trauma, tumour lysis syndrome, status epilepticus and some drugs; [14]) or by impairment of renal excretion. Urea-cycle enzymes can also become overwhelmed following gastrointestinal haemorrhage, urease-producing bacterial infections and the administration of some drugs [15].

Hyperammonaemia has been reported in veterinary patients in a variety of clinical settings. The most commonly reported causes are hepatic vascular anomalies [16,17] and hepatic failure, but blood ammonia elevation has also been described in association with congenital [18,19] and acquired urea-cycle disorders, including cobalamin deficiency in both dogs [20] and cats [21,22,23], and arginine deficiency in cats [24]. In cats, hyperammonaemia has also been described in azotaemic patients [25] and following seizure activity [26].

Regardless of whether or not clinical HE is apparent, hyperammonaemia has negative prognostic implications, and the duration of hyperammonaemia is positively correlated with long-term neurologic complications; thus, timely identification and treatment is essential [27,28]. With increasing recognition of hyperammonaemia in veterinary patients, the demand for newer and more readily available BA tests has increased. Historically, plasma ammonia has been measured by enzymatic or colorimetric assays that require transport of blood samples to in-house or reference laboratories [29]. Due to the labile nature of ammonium ions in plasma, careful sample handling, including rapid cooling and minimal exposure to air, is required, restricting their widespread use in the clinical setting [30]. The delay in result reporting also delays diagnosis, with inherent implications for patient outcomes [27]. Therefore, the use of POC devices to facilitate patient-side testing with the ability to provide instantaneous results offers many advantages.

Over the past 20 years, a POC blood ammonia assay (PocketChem^TM^ BA Analyser, Woodley Laboratory Diagnostics, Lancashire, UK) has been developed. Previous studies have confirmed this to be a viable option for the evaluation of BA in dogs and cats, suggesting good precision, acceptable linearity, and acceptable agreement with laboratory-based reference methods; however, the determination of appropriate cut-off values and the correlation of BA concentration with the severity of clinical signs was not concluded [31,32]. Indeed, clinicians currently use varying cut-off values for HE in dogs and cats based on personal clinical experience or human reference ranges.

The primary aim of the present study was to establish inferred reference intervals for BA in dogs and cats using the PocketChem^TM^ BA analyser by the comparison of patients with and without disease pathologies reported to cause BA elevation. A secondary aim was to document species differences between specific disease pathologies associated with hyperammonaemia.

## 2. Materials and Methods

### 2.1. Study Population

This was a single-centre, prospective, cross-sectional study using blood obtained from dogs and cats presenting to the internal medicine, neurology, surgery and first opinion departments of a multi-disciplinary veterinary hospital in the United Kingdom. Subject to written, informed owner consent, all dogs and cats presenting to the hospital were eligible for inclusion, regardless of signalment or disease pathology. However, compliant with ethical approval, BA measurement was performed only in patients undergoing blood sampling for diagnostic purposes in which surplus blood was available. Patients were excluded from the study in the absence of written informed owner consent, or if surplus blood or concurrent complete blood count and basic serum biochemistry results were not available. Further investigations, including additional blood work (including fasting and post-prandial serum bile acids and serum cobalamin) and imaging studies were at the discretion of the attending clinician.

### 2.2. Data Collection and Classification

Signalment, clinical history, and physical examination results were recorded for each patient along with laboratory test and diagnostic imaging results. Primary diagnosis and concurrent disease conditions were recorded based on the predominant reason for patient evaluation at the time of BA measurement. Primary diagnoses were grouped according to body system (hepatic, gastrointestinal, urinary, cardiorespiratory, neurological, other) and pathology type (inflammatory/infectious, neoplastic, other).

Azotaemia was defined as the presence of increased creatinine, increased urea, or both according to predefined laboratory reference ranges. Alanine aminotransferase (ALT) and alkaline phosphatase (ALP) were classified as elevated if they were >2× and >3× the upper limit of the predefined laboratory reference ranges, respectively. Where available, serum bilirubin and serum bile acids were considered to be elevated in the event of a result above the top end of the predefined laboratory reference range. Where serum cobalamin results were available, hypocobalaminaemia was defined as serum cobalamin <400 ng/dL according to Kather et al., 2020 [33]. Body condition was scored on a scale from 1 (underweight) to 9 (overweight), with suboptimal body condition score (BCS) being defined as 1–3/9.

Patients were additionally classified as having ‘liver disease’ (confirmed liver pathology) or ‘possible hepatic involvement’ (any patient with ALT, ALP, bile acid or bilirubin elevation) and/or azotaemia as defined above. The presence of ‘weight loss’, ‘gastrointestinal signs’ (vomiting and/or diarrhoea), and/or ‘central nervous system signs’ were assigned according to the clinical notes. Any recent seizure activity was documented along with its timing prior to BA measurement, and the presence/absence of urinary tract infection was noted. Finally, a label of ‘possible hepatic encephalopathy’ was assigned based on clinical suspicion of the attending clinician, as suggested by a retrospective review of the clinical notes; as such, all other possible causes of the neurological signs considered suggestive of HE were not necessarily excluded.

### 2.3. Ammonia Measurement

The PocketChem^TM^ BA analyser (measurement range 0–400 µg/dL) was used for BA measurement in all patients. All patients had been starved for >8 h prior to sampling. Whole blood was obtained via venipuncture of the jugular, cephalic, or saphenous vein and was placed directly into sample tubes as indicated for patient-specific diagnostic tests. A drop of any surplus blood was then immediately placed into the cap of a serum tube for BA measurement according to the manufacturer’s instructions. Briefly, a micropipette was used to transfer 20 µL of whole blood from the serum tube cap to the ammonia reagent strip. The strip was left to rest on a flat surface for 180 s and was then inserted into the PocketChem^TM^ BA reader. The ammonia concentration registered on the screen was documented.

### 2.4. Ethics Statement

The study design was approved by the ethics committee of the University of Nottingham, UK (ref: 3731 221207).

### 2.5. Data Manipulation and Statistical Analysis

Based on assessment of the clinical data as described above, patients were labelled according to whether or not they had disease pathology previously reported as a potential cause of BA elevation. Patients with conditions that could potentially result in BA elevation were further categorised into hepatic and non-hepatic aetiologies. Patients in the hepatic group included those with ALT, ALP, bilirubin, and/or bile acid elevation, regardless of whether or not liver disease was confirmed. The non-hepatic group included patients with azotaemia, hypocobalaminaemia, gastrointestinal bleeding, or urinary tract infection in the absence of hepatic parameter abnormalities, those with inherited errors of metabolism, and those that had received blood transfusions. Patients in which all known causes of hyperammonaemia could be excluded were used to establish inferred reference ranges, and BA concentrations of patients in which BA elevation was possible were compared against these ranges.

Following the visualisation of Q-Q plots and Shapiro–Wilk testing, continuous BA concentration data, age, and BCS were considered as non-parametric data and were reported as median, interquartile range (IQR), and range. Comparisons with categorical clinical subgroups were carried out using the Mann–Whitney test. Additionally, age (dogs: <4 y, 4–8 y, >8 y; cats: <3 y, 3–10 y, >10 y), BCS (1–3, 3–6, 7–9), and BA (detectable/BA > 0 versus undetectable/BA = 0) were categorised and compared between clinical subgroups using the Pearson Chi^2^ test. Multivariable analysis was performed using a Quade non-parametric analysis of covariance with Bonferroni adjustment of significance to account for pairwise comparisons. Significance was set at *p* < 0.05.

## 3. Results

### 3.1. Descriptive Statistics (Dogs)

Blood ammonia concentration results from 175 dogs were available for inclusion, of which 93 (53.1%) were male (68 neutered and 25 entire) and 82 (46.9%) were female (65 neutered and 17 entire). The median age was 7 years 4 months (range 5 months to 15 years). The breed distribution included 38 (21.7%) cross-breed dogs, 17 (9.7%) Labrador Retriever, 10 (5.7%) Shih Tzu, 8 (4.6%) Border Collie, 5 (2.9%) each for Golden Retriever, Cocker Spaniel, Boxer and Border Terrier, 4 (2.3%) each for Miniature Schnauzer and Miniature Pinscher, 3 (1.7%) each for Whippet, Greyhound, Miniature Poodle, and Doberman. The remaining 90 dogs comprised 44 different breeds, each represented by one or two dogs. Body condition score was available for 158 dogs, of which the median was 5/9 (range of 1 to 8). Information regarding weight loss was available in 145 dogs, of which recent weight loss was reported in 68 (47%). Affected body systems were as follows: 32 (18.3%) hepatic, 48 (27.4%) gastrointestinal, 19 (10.9%) cardiorespiratory, 17 (9.7%) neurological, 16 (9.1%) urinary, 41 (23.4%) other, 2 (1.1%) unknown. Gastrointestinal signs were reported in 57 dogs and lower urinary tract signs in 14 dogs, of which 6 had positive urine cultures. Central nervous system signs were reported in 29 dogs, 10 with recent seizure activity and 10 with clinically suspected HE. The breakdown of disease pathology was as follows: 77 (44.0%) inflammatory/infectious, 40 (22.9%) neoplastic, 50 (28.6%) other. The disease pathology type was undefined in seven dogs and one dog was healthy. Azotaemia was present in 25 (14.3%) dogs. Serum cobalamin measurement was available in 44 dogs, of which 23 (52.3%) had B12 deficiency.

Of the 175 dogs included in the study, 124 (71%) had undetectable blood ammonia (BA = 0). Concentrations of blood ammonia in dogs in which it was detectable (BA > 0) ranged from 7 µg/dL to 400 µg/dL as follows: 1–10 µg/dL (n = 9), 11–20 µg/dL (n = 22), 21–30 µg/dL (n = 5), 31–60 µg/dL (n = 5), 61–100 µg/dL (n = 4), >100 µg/dL (n = 7). The median BA concentration was 15 µg/dL (range 0–400). Disease categories of dogs with detectable BA comprised hepatic (n = 20, BA 7–400 µg/dL), gastrointestinal (n = 12, BA 7–38 µg/dL), neurological (n = 6, BA 10–20 µg/dL), urinary (n = 4, BA 11–26 µg/dL), and other (n = 9, BA 7–29 µg/dL). All dogs with BA > 40 µg/dL had liver disease. Only one dog without liver disease had BA > 30 µg/dL; this dog had hypoadrenocorticism with gastrointestinal bleeding. All dogs with a clinical suspicion of HE and all dogs with hepatic vascular anomalies (congenital or acquired) had BA > 40 µg/dL.

Following the exclusion of all dogs with evidence to support possible hepatic involvement (confirmed liver disease, ALT or ALP elevation, bile acid elevation, and/or hyperbilirubinaemia), 26/116 dogs had detectable BA (median 14 µg/dL, IQR 6 µg/dL, range 7–38 µg/dL). These comprised dogs with gastrointestinal disease (n = 9/35), neurological disease (n = 6/14), cardiorespiratory disease (n = 3/12), urinary tract disease (n = 4/13), musculoskeletal disorders (n = 1/6), endocrinopathies (n = 1/3), and haematologic disease (n = 2/5). Only 1 (the dog with hypoadrenocorticism and gastrointestinal bleeding) of these 26 dogs had BA > 30 µg/dL. Following the additional exclusion of all dogs with other potential causes of hyperammonaemia (azotaemia, hypocobalaminaemia, gastrointestinal bleeding, urinary tract infection), 13/82 dogs had detectable BA (median 13 µg/dL, IQR 5 µg/dL, range 9–29 µg/dL). These comprised dogs with gastrointestinal disease (n = 3/22), neurological disease (n = 3/14), cardiorespiratory disease (n = 3/12), urinary tract disease (n = 3/10), musculoskeletal disorders (n = 1/6), endocrinopathies (n = 0/2), and haematologic disease (n = 0/3).

### 3.2. Blood Ammonia Comparisons (Dogs)

There was no difference in continuous age or BCS data between dogs with detectable and undetectable BA (Table 1a), and no difference in the distribution of BA concentration between the age group and BCS categories (Table 1b). Blood ammonia was detectable more frequently in male than in female dogs (*p* = 0.021, OR 2.203), and in those with liver pathology (*p* < 0.001, OR 4.540), central nervous system signs (*p* > 0.001, OR 4.808) and azotaemia (*p* = 0.028, OR 2.580) (Table 2). When assessed as a continuous variable, BA was also significantly higher in male than in female dogs (*p* = 0.022), and in those with liver pathology (*p* < 0.001) and central nervous system signs (*p* < 0.001). The difference in BA concentration between azotaemic and non-azotaemic dogs was approaching, but did not reach, statistical significance in univariable analysis (*p* = 0.054) (Table 2). The presence of weight loss, gastrointestinal signs, and cobalamin deficiency did not impact whether or not BA was detectable (Table 2), and there was no difference in BA concentration between these subgroups (Table 3). Similarly, no difference was found between dogs with inflammatory/infectious disease and those with neoplasia (Table 2 and Table 3). In multivariable analysis, detectable BA remained more likely in male dogs (*p* = 0.031), and in dogs with hepatic involvement (*p* = 0.001), CNS signs (*p* < 0.001), and azotaemia (*p* = 0.010) (Table 4). When assessed as a continuous variable, the distribution of BA concentration also remained significantly different between male and female dogs (*p* = 0.041) and between dogs with and without liver disease (*p* = 0.027), hepatic involvement (*p* < 0.001), CNS signs (*p* < 0.001) and azotaemia (*p* = 0.024) (Table 4).

### 3.3. Descriptive Statistics (Cats)

Blood ammonia concentration results from 63 cats were available for inclusion, of which 40 (63.5%) were male (37 neutered and 3 entire) and 23 (36.5%) were female (21 neutered and 2 entire). The median age was 8 years 3 months (range 5 months to 17 years). The breed distribution included 41 (65%) domestic shorthair, 6 (9.5%) domestic longhair, 4 (6.3%) Maine Coons, 3 (4.8%) Siamese, 2 (3.2%) British Shorthair, and 1 each of Bengal, Birman, Burmese, Oriental Shorthair, Persian, Russian Blue and Sphinx. Body condition score was available for 61 cats, of which the median was 4/9 (range of 2 to 9). Information regarding weight loss was available in 61 cats of which recent weight loss was reported in 30 (49.2%). Affected body systems were as follows: 21 (33.3%) gastrointestinal, 16 (25.4%) hepatic, 8 (12.7%) urinary, 8 (12.7%) respiratory, 4 (6.3%) neurological, 3 (4.8%) endocrine, 2 (3.2%) other, 1 (1.6%) unknown. Gastrointestinal signs were reported in 30 cats and lower urinary tract signs in four cats. Only one cat had a positive urine culture. Central nervous system signs were reported in seven cats, three with recent seizure activity and five with suspected HE. The breakdown of disease pathology was as follows: 35 (55.6%) inflammatory/infectious, 13 (20.6%) neoplastic, 14 (22.2%) other. The disease pathology type was undefined in one cat. Azotaemia was present in 23 (36.5%) cats. Serum cobalamin measurement was available in 23 cats, of which 8 (34.8%) had B12 deficiency.

Of the 63 cats included in the study, 29 (46%) had undetectable blood ammonia (BA = 0). Concentrations of blood ammonia in cats in which it was detectable (BA > 0) ranged from 7 µg/dL to 267 µg/dL as follows: 1–10 µg/dL (n = 4), 11–20 µg/dL (n = 12), 21–30 µg/dL (n = 7), 31–40 µg/dL (n = 4), 41–60 µg/dL (n = 2), 61–100 µg/dL (n = 2), > 100 µg/dL (n = 3). The median BA concentration was 7.0 µg/dL (range 0–267). Disease categories of cats with detectable BA comprised gastrointestinal (n = 13, 7–61 µg/dL), hepatic (n = 10, 7–264 µg/dL), urinary (n = 3, 11–77 µg/dL) endocrine (n = 2, 14–21 µg/dL), cardiorespiratory (n = 2, 15–49 µg/dL), neurological (n = 2, 7–23 µg/dL), and other (n = 3, 13–267 µg/dL). The one cat with a positive urine culture had undetectable BA. Five cats had a clinical suspicion of HE with BA ranging from 21 to 267 µg/dL. Of the three cats with markedly elevated BA (>100 µg/dL), one had a portosystemic shunt (BA 264 µg/dL), one had a congenital urea cycle abnormality (BA 267 µg/dL), and one had feline infectious peritonitis with liver pathology, hypocobalaminaemia, and weight loss/sarcopenia (BCS 2/9) (102 µg/dL). All three had clinically suspected HE.

Following the exclusion of all cats with evidence to support possible hepatic involvement (confirmed liver disease, ALT or ALP elevation, bile acid elevation, and/or hyperbilirubinaemia), 23/43 cats had detectable BA (median 16 µg/dL, IQR 26 µg/dL, range 7–267 µg/dL). These comprised cats with gastrointestinal disease (n = 12/19), neurological disease (n = 3/3), cardiorespiratory disease (n = 3/7), urinary tract disease (n = 2/8), endocrinopathies (n = 1/3), and other (n = 2/2). Following the additional exclusion of cats with other potential causes of hyperammonaemia (azotaemia, hypocobalaminaemia, urinary tract infection, inherited errors of metabolism, and weight loss), 9/19 cats had detectable BA (median 13 µg/dL, IQR 9 µg/dL, range 7–25 µg/dL). These comprised cats with gastrointestinal disease (n = 4/8), neurological disease (n = 2/2), cardiorespiratory disease (2/5), and one cat with uveitis. The remaining 2/19 cats had urinary tract disease, and both had undetectable BA.

### 3.4. Blood Ammonia Comparisons (Cats)

There was no difference in continuous age or BCS data between cats with detectable and undetectable BA (Table 5a) and no difference in the distribution of BA concentration between age group and BCS categories (Table 5b).

The presence of weight loss, gastrointestinal signs and cobalamin deficiency did not impact whether or not BA was detectable (Table 6), and there was no difference in the distribution of BA concentration data between these subgroups (Table 7). Similarly, no differences were found between cats with inflammatory/infectious disease and those with neoplasia (Table 6 and Table 7). In multivariable analysis independent of weight loss, liver disease, azotaemia and CNS signs, no differences were found in any of the examined clinical parameters between cats with and without detectable BA, nor was there any difference in the distribution of continuous BA concentration data across the clinical subgroups (Table 8).

## 4. Discussion

This study aimed to establish preliminary inferred reference ranges for BA concentration in dogs and cats using a POC device, via the comparison of patients with and without pathologies reported to be associated with BA elevation. Once patients with possible causes of BA elevation had been excluded, all remaining dogs and cats had BA < 30 µg/dL and <25 µg/dL, respectively, suggesting that these might represent appropriate upper cut-off limits to define normal BA concentration. Since many dogs and cats had undetectable BA, the inferred reference ranges were determined to be 0–30 µg/dL and 0–25 µg/dL, respectively. These reference ranges are lower than those suggested for humans, where lower and upper limits variably range from 11 µg/dL to 61 µg/dL [7]. Whether this represents inherent species differences or suboptimal sensitivity of the PocketChem^TM^ BA methodology is uncertain, but the large number of dogs and cats with undetectable BA raises concern for the latter. Unfortunately, in the absence of a comparative reference method, an assessment of sensitivity or specificity was outside the scope of this study. However, since only the upper (rather than lower) reference limit is considered clinically important, the PocketChem^TM^ BA device is nevertheless considered sensitive enough to establish the study aims. In a previous study, the PocketChem^TM^ BA device was compared with an enzymatic assay reference method in 38 dogs and 4 cats with suspected HE [31]. BA concentrations were <100 µg/dL in only 11.1% of the study population and no patients had BA < 7 µg/dL; however, patients were recruited to the study on the basis of suspected HE. Thus, the high number of undetectable results in the current study is likely explained by the predominant inclusion of patients expected to have normal BA concentrations. The alternative explanation that the PocketChem^TM^ BA device was randomly generating results of zero seems unlikely given its previous validation. Since all samples in the current study were tested immediately post-collection, sample delay was excluded as a cause of undetectable results, with a previous study having concluded a delay of 2–3 min at ambient temperature to be acceptable [32]. Thus, despite a perceived lack of sensitivity, the use of the PocketChem^TM^ BA device to distinguish abnormal from normal BA is considered clinically applicable, and undetectable results should be considered normal. The disparity in proportion of undetectable results between dogs (71%) and cats (46%) also suggests that normal BA concentrations may generally be lower in dogs than in cats. The alternative possibility that the methodology is less sensitive in cats is less likely, considering the proportion of cats with high BA.

The previous study, comprising dogs and cats with suspected HE, also suggested that a higher cut-off of >60 µmol/L may be appropriate for the detection of HE [31]. However, in comparison with the current study in which only 9.1% (16/175) of dogs and 17.5% (11/63) of cats had suspected BA elevation, 72.2% of the study population had high BA. Indeed, while the previous study sought to identify a lower limit for the detection of clinically apparent HE, the current study attempted to define an upper limit for normal BA concentration; thus, the results are not directly comparable. Considering its importance in humans, subclinical HE in dogs and cats is likely to be a poorly recognised syndrome, with the lack of early detection and treatment likely leading to clinically important sequalae [27,28]. The establishment of upper normal reference limits as well as lower limits for the diagnosis of overt HE is therefore considered to have clinical value.

In accordance with the previous literature in both dogs [16] and humans [34], the majority of dogs (13/14, 93%) with BA exceeding the inferred cut-off had liver disease. In the remaining dog, hyperammonaemia was attributed to gastrointestinal bleeding, another well-documented inciting factor for encephalopathy [2]. Despite previous associations with hyperammonaemia in humans [15,35] and in one dog [36], no dogs with azotaemia or urinary tract infection had BA > 30 µg/dL. Independent of the other variables, BA was more likely to be detectable in male dogs, as well as those with hepatic involvement, neurological signs, and azotaemia, and the distribution of BA concentration was different across these subcategories. In both humans and rodents, an influence of testosterone on renal ammonia metabolism has been proven [37], and these results suggest the possibility of a similar mechanism in dogs. Similarly, since renal metabolism of ammonia is well recognised across all species, higher BA values are not unexpected in the context of azotaemia [25,38]. In humans, BA concentration is significantly higher in neonates but otherwise has no particular age correlation [7]. No dogs or cats in the current study were under five months of age, so the lack of age association is not unexpected. While the large number of dog breeds prohibited any assessment of genetic variation, differences in the frequency and magnitude of BA elevation between human ethnic groups are largely attributed to social attitudes towards alcohol consumption rather than to race [7].

In contrast with the dog population, no associations were found between signalment, clinical signs, or disease categories amongst the 63 cats, and only 6/12 (50%) cats with BA exceeding the inferred upper reference limit had hepatic disease. Thus, while hyperammonaemia is likely to be a good predictor of liver disease in dogs, high BA appears to be associated with a much wider variety of disease pathologies in cats. In human medicine, non-cirrhotic hyperammonaemia is a rare but increasingly recognised phenomenon [34,39,40,41]. The term is used to encompass all causes of hyperammonaemia independent of liver failure, including not only portosystemic vascular anomalies, but also a wide variety of other pathologies, ranging from primary and secondary urea-cycle disorders to haematologic malignancies, cardiovascular disease, and pulmonary disease. The findings of the current study, coupled with previous reports describing varying causes of hyperammonaemia in cats [25,26], suggest that this species may be particularly prone to BA elevation. Of the 6/12 cats with BA > 25 µg/dL and no evidence of liver disease, one had a BA concentration of 267 µg/dL and was diagnosed with hyperornithemia–hyperammonemia–homocitrullinuria, a syndrome expected to cause marked BA elevation [42]. Two of the six cats had renal disease, a well-documented cause of BA elevation, albeit with multifactorial mechanisms [25]; one of these cats had acute kidney injury and ureteral obstruction (BA 77 µg/dL) and the other had advanced chronic kidney disease and haemoabdomen (BA 39 µg/dL). In the remaining three cats, exclusion from the inferred reference range was applied based on weight loss, but the true cause of hyperammonaemia was less well defined. Two of the three had weight loss with suboptimal BCS (3/9), and had diseases commonly associated with a catabolic state: namely, small-cell alimentary lymphoma (BA 61 µg/dL) and chronic enteropathy (BA 44 µg/dL). The third cat had a BA of 49 µg/dL but, although convincing weight loss was documented, the cat’s body condition remained good (5/9). The cat was diagnosed with a nasopharyngeal polyp and chronic lower airway disease, but since diagnostic imaging was limited to the head and thorax, concurrent disease could not be excluded.

In addition to the liver, skeletal muscle contributes to the regulation of BA levels by absorbing ammonia from plasma and releasing glutamine. In the face of liver failure, this mechanism becomes more heavily relied upon; thus, HE is potentiated by muscle atrophy [7]. As a breakdown product of amino acid metabolism, the rate of ammonia production is also expected to increase during catabolic states; thus, sarcopenia has a well-documented association with HE in humans [43]. Catabolism has been associated with altered ammonia metabolism in dogs [44] and of outright hyperammonaemia in humans following Roux-En-Y Gastric Bypass surgery [45]. While catabolism is therefore a plausible cause of hyperammonaemia in the three cats described above, it is notable that weight loss and poor body condition scores were reported in many other cats that had undetectable BA or values below the inferred cut-off. Further investigations in a population of cats with diseases leading to a catabolic state are therefore necessary to ascertain whether weight loss and/or sarcopenia are clinically important causes of hyperammonaemia in cats. An evaluation of plasma amino acid profiles would also help to provide information on the origin of hyperammonaemia. An alternative explanation for the wide variety of diseases associated with higher BA in cats is that the inferred upper reference limit is too low, such that some cats excluded from the inferred reference range for having potential causes of hyperammonaemia in fact had normal BA. Indeed, the higher proportion of cats with detectable BA (54%) in comparison with dogs (29%) raises the possibility of higher normal values in this species.

The clinical relevance of the magnitude of hyperammonaemia in cats and dogs also remains unclear. Whilst 8/10 dogs with suspected HE had BA > 60 µg/dL, 2 had somewhat lower values (44 µg/dL and 47 µg/dL). Similarly, while three of the four cats with a clinical suspicion of hepatic encephalopathy had BA > 100 µg/dL, one had a relatively low value (31 µg/dL), albeit still outside the inferred reference range. This cat was diagnosed with portal hypertension and Budd–Chiari-like syndrome secondary to hepatic carcinoma invading the portal vein and caudal vena cava. The diagnosis of HE in this patient was reached based on the presence of antibiotic responsive mental obtundation and non-lateralised neurological deficits compatible with a forebrain lesion. The aetiology of HE is multifactorial and clinical signs do not always correlate with the degree of hyperammonaemia; thus, the diagnosis of HE relies on the interpretation of clinical signs and response to treatment as well as laboratory results [2]. Further studies with more stringent inclusion criteria necessitating standardised diagnostic investigations, clinical and neurological evaluations, and follow-up are required to further evaluate the clinical implications associated with the magnitude of BA elevation.

This study has several limitations; first and foremost, the disproportionate number of dogs and cats with undetectable BA resulted in unequal group sizes with relatively low numbers within the higher BA ranges. Other than a comparison of animals with detectable versus undetectable BA, this precluded direct statistical comparisons based on BA categorisation. In particular, statistical comparisons of clinical parameters between animals with BA above and below the inferred cut-off values would have been ideal (i.e., <30 µg/dL versus >30 µg/dL and <25 µg/dL versus >25 µg/dL for dogs and cats, respectively). Similarly, the lack of inclusion of healthy animals risks the possibility that some dogs and cats within the inferred reference ranges might have non-clinically significant BA elevations rather than truly being within normal limits for a healthy population. Thus, confirmation of appropriate population-wide reference ranges for dogs and cats requires larger studies including healthy animals and using established guidelines (such as those given by the American Society for Veterinary Clinical Pathology) for the development of reference intervals in healthy animals. Another important limitation was the reliance on clinical notes for the documentation of clinical signs—most importantly, the presence or absence of weight loss and clinical signs consistent with HE. The consistent and accurate recording of clinical signs by attending clinicians and the interpretation of clinical notes by the authors cannot be absolutely guaranteed. Finally, diagnostic investigations undertaken in the study population were not standardised. Clinical decision making was solely at the discretion of the attending clinician; thus, not all dogs and cats underwent exhaustive investigations to exclude occult causes of hyperammonaemia or to confirm a clinical suspicion of HE. In particular, not all patients had imaging of the abdomen, and very few had triple-phase CT angiography, which is considered the most sensitive non-invasive technique for the diagnosis of hepatic vascular anomalies [46]. Nevertheless, this imaging modality was undertaken in all patients in which hepatic vascular anomalies were considered a clinically relevant differential diagnosis, and it is unlikely that significant liver dysfunction was under-interpreted in our study population given the strict criteria used to define possible hepatic involvement.

In summary, this study provides preliminary ‘inferred’ reference ranges based on comparisons between dogs and cats with and without disease pathologies that have been reported to cause BA elevation. Further studies in healthy animals and in larger populations of dogs and cats with hyperammonaemia are required to further define true reference ranges, and to establish sensitivity and specificity data for the detection of HE in dogs and cats.

## 5. Conclusions

The results suggest an inferred upper reference limit of 30 µg/dL and 25 µg/dL for BA in dogs and cats, respectively. The proportion of dogs with detectable BA was much lower than that of cats, suggesting the possibility of lower basal BA concentrations in this species. Whilst BA > 30 µg/dL appears to be strongly predictive of liver disease in dogs, BA > 25 µg/dL was associated with a variety of disease pathologies in cats. Additional research using larger cohorts of healthy as well as diseased dogs and cats should be aimed at confirming these inferred reference ranges, further characterising species differences, and elucidating the clinical implications for varying magnitudes of BA elevation.

## Figures and Tables

**Table 1:** (**a**) Comparisons of age and body-condition-score data between dogs with undetectable (0) and detectable (>0) blood ammonia (**b**) Comparisons of continuous blood ammonia concentration data between age and body-condition-score categories in dogs.

**(a)**							
***Clinical Feature***	***Binary BA Subgroup***	***n***	***Median BA*** ***(IQR)***	***Range*** ***(Min-Max)***	***U***	***SE***	***p*** ***(2-Sided)***
Age (y)	0	124	7.96 (5.64)	14.42 (0.58–15.00)	2915	304.526	0.417
	>0	51	6.83 (5.25)	13.41 (0.42–13.83)			
BCS (1–9/9)	0	116	5 (2)	7 (1–8)	2110	239.380	0.263
	>0	41	3 (2)	5 (3–8)			
**(b)**							
***Clinical feature***	***Subgroup***	***n***	***Median*** ***BA(IQR)***	***Range*** ***(min-max)***	***H***	***df***	***p*** ***(2-sided)***
Age (y)	<4	39	0 (11)	0–139	2.309	2	0.315
	4–8	52	0 (16)	0–171			
	>8	84	0 (7)	0–400			
BCS (1–9/9)	1–3	17	0 (15)	0–96	1.071	2	0.585
	4–6	99	0 (7)	0–400			
	7–9	41	0 (4)	0–135			

BCS = body condition score; y = years; BA = blood ammonia; n = number of dogs; IQR = interquartile range; U = Mann–Whitney test statistic; SE = standard error; *p* = significance at *p* < 0.05. H = Kruskal–Wallis test statistic; df = degrees of freedom.

**Table 2:** Comparisons of clinical parameter subgroups between dogs with undetectable (0) and detectable (>0) blood ammonia.

*Clinical Feature*	*Binary BA Subgroup*	*n:* *BA Subgroup*	*n (%):* *Clinical Feature*	*t*	*df*	*p* *(2-Sided)*	*OR*	*95% CI*
Male sex	0	124	59 (47.6)	5.286	1	**0.021**	2.203	1.116–4.352
	>1	51	34 (66.7)					
Weight loss	0	104	47 (45.2)	0.429	1	0.512	-	-
	>0	41	2 (51.2)					
*^1^ Liver disease	0	114	34 (29.8)	5.652	1	**0.017**	2.262	1.146–4.466
	>0	51	25 (49.0)					
*^2^ Hepatic involvement	0	122	18 (14.8)	16.996	1	**<0.001**	4.540	2.154–9.608
	0	50	22 (44)					
GI signs	0	124	41 (33.1)	0.047	1	0.828	-	-
	>0	52	16 (31.4)					
CNS signs	0	124	12 (9.7)	15.177	1	**<0.001**	4.808	2.087–11.079
	>0	50	17 (34%)					
Azotaemia	0	122	13 (10.7)	4.821	1	**0.028**	2.580	1.086–6.131
	>0	51	12 (23.5)					
B12 deficiency	0	31	14 (45.2)	2.127	1	0.145	-	-
	>0	13	9 (69.2)					
Inflammatory pathology	0	90	61 (80.3)	0.908	1	0.341	-	-
	>0	26	15 (19.7)					

BA = blood ammonia; n = number of dogs; t = Chi^2^ test statistic; df = degrees of freedom; CI = confidence interval; *p* = significance at *p* < 0.05, *^1^ = dogs with confirmed liver disease; *^2^ = dogs with raised liver enzymes, bile acids, or bilirubin.

**Table 3:** Comparisons of continuous blood ammonia data between clinical parameter subgroups in dogs.

*Clinical Feature*	*Subgroup*	*n*	*Median BA (IQR)*	*Range*	*U*	*df*	*SE*	*t*	*p (2-Sided)*
Sex	Male	93	0 (14)	0–171	4429.5	1	268.421	2.297	**0.022**
	Female	82	0 (0)	0–400					
Weight loss	No	77	0 (9)	0–135	2748.0	1	200.282	0.648	0.517
	Yes	68	0 (9)	0–400					
*^1^ Liver disease	No	106	0 (2)	0–38	3847.5	1	240.777	2.992	**0.003**
	Yes	59	0 (24)	0–400					
*^2^ Hepatic involvement	No	132	0 (0)	0–38	3693.0	1	221.249	4.759	**<0.001**
	Yes	40	9 (61)	0–400					
GI signs	No	118	0 (10)	0–400	3310.5	1	252.085	−0.208	0.835
	Yes	57	0 (11)	0–107					
CNS signs	No	145	0 (0)	0–171	3147.5	1	202.728	4.797	**<0.001**
	Yes	30	11.5 (84)	0–400					
Azotaemia	No	148	0 (9)	0–171	2209.0	1	186.631	1.924	0.054
	Yes	25	0 (14)	0–400					
B12 deficiency	No	21	0 (0)	0–21	296.0	1	34.313	1.588	0.112
	Yes	23	0 (15)	0–44					
Pathology type	Inflammatory	76	0 (0)	0–400	1606.5	1	125.685	0.688	0.491
	Neoplastic	40	0 (9)	0–28					

n = number of dogs; BA = blood ammonia; IQR = interquartile range; U = Mann–Whitney test statistic; df = degrees of freedom; SE = standard error; t = standard test statistic; *p* = significance at *p* < 0.05, *^1^ dogs with confirmed liver disease; *^2^ dogs with raised liver enzymes, bile acids, or bilirubin.

**Table 4:** Comparisons of binary (undetectable versus detectable) and continuous blood ammonia concentration data between clinical parameter subgroups in dogs independent of sex, liver disease, CNS signs, and azotaemia.

*Clinical Feature*	*BA Data* *Format*	*F*	*t*	*dfh*	*df*	*p*
Age (<4 y/4–8 y/>8 y)	Binary	0.186	-	2	161	0.830
	Continuous	0.472	-	2	161	0.625
Sex (male/female)	Binary	4.752	−2.180	1	162	**0.031**
	Continuous	4.236	−2.058	1	162	**0.041**
Weight loss (y/n)	Binary	0.072	−0.269	1	135	0.788
	Continuous	0.075	−0.275	1	135	0.784
BCS (1–3, 4–6, 7–9)	Binary	0.433	-	2	146	0.649
	Continuous	0.569	-	2	146	0.567
Cobalamin deficiency (y/n)	Binary	1.987	−1.410	1	42	0.166
	Continuous	2.551	−1.597	1	42	0.118
Pathology type (inflammatory/neoplastic)	Binary	0.659	−0.811	1	108	0.419
	Continuous	0.243	−0.493	1	108	0.623
*^1^ Liver disease (y/n)	Binary	2.297	−1.510	1	162	0.133
	Continuous	5.000	−2.236	1	162	**0.027**
*^2^ Hepatic involvement (y/n)	Binary	11.814	−3.437	1	168	**0.001**
	Continuous	17.599	−4.195	1	168	**<0.001**
Azotaemia (y/n)	Binary	6.887	−2.624	1	162	**0.010**
	Continuous	5.195	−2.279	1	162	**0.024**
GI signs (y/n)	Binary	0.255	−0.505	1	162	0.614
	Continuous	0.600	−0.775	1	162	0.440
CNS signs (y/n)	Binary	16.924	−4.114	1	162	**<0.001**
	Continuous	25.161	−5.016	1	162	**<0.001**

BA = blood ammonia; BCS = body condition score; F = ANOVA ratio of variances; t = test statistic; dfh = hypothesis degrees of freedom; df = degrees of freedom; *p* = significance at *p* < 0.05); *^1^ dogs with confirmed liver disease; *^2^ dogs with raised liver enzymes, bile acids, or bilirubin.

**Table 5:** (**a**) Comparisons of age and body-condition-score data between cats with undetectable (0) and detectable (>0) blood ammonia. (**b**) Comparisons of continuous blood ammonia concentration data between age and body-condition-score categories in cats.

**(a)**							
***Clinical Feature***	***Binary BA Subgroup***	***n***	***Median*** ***(IQR)***	***Range*** ***(Min-Max)***	***U***	***SE***	***p*** ***(2-Sided)***
Age (y)	0	29	6.67 (8.3)	16.6 (0.4–17.0)	522.0	72.504	0.783
	>0	34	9.0 (7.0)	14.6 (0.4–15.0)			
BCS (1–9/9)	0	28	5 (2)	6 (2–8)	423.0	67.386	0.563
	>0	33	4 (3)	7 (2–9)			
**(b)**							
***Clinical feature***	***Subgroup***	***n***	***Median BA*** ***(IQR)***	***BA range*** ***(min-max)***	***H***	***df***	***p*** ***(2-sided)***
Age (y)	<3	14	0 (24)	0–264	0.585	2	0.747
	3–10	24	4.5 (25)	0–49			
	>10	25	11 (20)	0–267			
BCS (1–9/9)	1–3	25	12 (38)	0–267	2.443	2	0.295
	4–6	28	8 (21)	0–49			
	7–9	8	0 (12)	0–25			

BCS = body condition score; y = years; BA = blood ammonia; n = number of cats; IQR = interquartile range; U = Mann–Whitney test statistic; SE = standard error; *p* = significance at *p* < 0.05. H = Kruskal–Wallis test statistic; df = degrees of freedom.

**Table 6:** Comparisons of clinical parameter subgroups between cats with undetectable (0) and detectable (>0) blood ammonia.

*Clinical Feature*	*Binary BA Subgroup*	*n (%) with Clinical Feature*	*t*	*df*	*p* *(2-Sided)*
Male sex	0	21/29 (72.4)	1.845	1	0.174
	>0	19/34 (55.9)			
Weight loss	0	12/28 (42.9)	0.828	1	0.363
	>0	18/33 (54.5)			
Liver pathology	0	9/29 (31.0)	0.037	1	0.847
	>0	11/33 (33.3)			
GI signs	0	16/29 (55.2)	0.229	1	0.268
	>0	14/34 (41.2)			
CNS signs	0	2/29 (6.9)	0.966	1	0.326
	>0	5/34 (14.7)			
Lower urinary tract signs	0	3/29 (10.3)	1.443	1	0.230
	>0	1/34 (2.9)			
Azotaemia	0	12/28 (42.9)	0.726	1	0.394
	>0	11/34 (32.4)			
Cobalamin deficiency	0	2/9 (22.2)	1.028	1	0.311
	>0	6/14 (42.9)			
Inflammatory pathology	0	18/23 (78.3)	0.639	1	0.424
	>0	17/25 (68.0)			

BA = blood ammonia; n = number of cats; t = Chi^2^ test statistic; df = degrees of freedom; *p* = significance at *p* < 0.05.

**Table 7:** Comparisons of continuous blood ammonia data between clinical parameter subgroups in cats.

*Clinical Feature*	*Subgroup*	*n*	*Median BA (IQR)*	*BA Range*	*U*	*df*	*SE*	*t*	*p (2-Sided)*
Sex	Male	23	0 (22)	0–267	247.0	1	66.533	−0.496	0.620
	Female	40	9 (19)	0–264					
Weight loss	No	31	0 (16)	0–267	540.5	1	65.869	1.146	0.252
	Yes	30	11.5 (31)	0–102					
*^1^ Liver disease	No	47	7 (16)	0–267	453.0	1	60.152	1.280	0.201
	Yes	16	16.5 (31)	0–264					
*^2^ Hepatic involvement	No	42	7 (17)	0–267	456.5	1	62.907	0.580	0.562
	Yes	20	9 (29)	0–264					
GI signs	No	33	13 (27)	0–267	397.0	1	69.018	−1.420	0.156
	Yes	30	0 (17)	0–61					
CNS signs	No	56	7 (20)	0–77	270.0	1	43.430	1.704	0.080
	Yes	7	23 (264)	0–267					
Azotaemia	No	39	9 (23)	0–267	404.0	1	65.376	−0.681	0.496
	Yes	23	0 (19)	0–77					
B12 deficiency	No	15	9 (44)	0–267	71.0	1	15.022	0.732	0.464
	Yes	8	14 (53)	0–102					
Pathology type	Inflammatory	35	0 (21)	0–102	255.5	1	40.656	0.689	0.491
	Neoplastic	13	14 (20)	0–61					

BA = blood ammonia; n = number of cats; IQR = interquartile range; U = Mann–Whitney test statistic; df = degrees of freedom; SE = standard error; t = standard test statistic; *p* = significance at *p* < 0.05; *^1^ cats with confirmed liver disease; *^2^ cats with raised liver enzymes, bile acids, or bilirubin.

**Table 8:** Comparisons of binary (undetectable versus detectable) and continuous blood ammonia concentration data between clinical parameter subgroups in cats independent of weight loss, liver disease, CNS signs, and azotaemia.

*Clinical Feature*	*BA Data Format*	*F*	*t*	*dfh*	*df*	*p*
Age (<3 y/3–10 y/>10 y)	Binary	0.930	-	2	57	0.400
	Continuous	0.256	-	2	57	0.775
Sex (male/female)	Binary	2.799	1.673	1	58	0.100
	Continuous	0.301	0.549	1	58	0.585
Weight loss (y/n)	Binary	0.602	−0.776	1	58	0.441
	Continuous	0.909	−0.954	1	58	0.344
BCS (1–9/9)	Binary	0.481	-	2	55	0.621
	Continuous	0.561	-	2	55	0.574
Cobalamin deficiency (y/n)	Binary	0.582	−0.763	1	20	0.454
	Continuous	0.249	−0.499	1	20	0.623
Pathology type (inflammatory/neoplastic)	Binary	0.005	−0.069	1	43	0.945
	Continuous	0.000	−0.018	1	43	0.986
*^1^ Liver disease (y/n)	Binary	0.022	−0.149	1	58	0.820
	Continuous	0.271	−0.520	1	58	0.605
*^2^ Hepatic involvement (y/n)	Binary	0.612	0.782	1	57	0.437
	Continuous	0.881	0.938	1	57	0.352
Azotaemia (y/n)	Binary	0.682	0.826	1	58	0.412
	Continuous	0.356	0.596	1	58	0.553
GI signs (y/n)	Binary	0.820	0.906	1	58	0.369
	Continuous	1.180	1.086	1	58	0.282
CNS signs (y/n)	Binary	0.968	−0.984	1	58	0.329
	Continuous	3.190	−1.786	1	58	0.079

BCS = body condition score; BA = blood ammonia; F = ANOVA ratio of variances; t = test statistic; dfh = hypothesis degrees of freedom; df = degrees of freedom; *p* = significance at *p* < 0.05; *^1^ cats with confirmed liver disease; *^2^ cats with raised liver enzymes, bile acids, or bilirubin.

## Data Availability

The original data presented in the study are available in Mendelay Data at DOI 10.17632/k5c4rhvsh5.1 (Available online: https://data.mendeley.com/datasets/k5c4rhvsh5/1 (accessed on 2 May 2025)).

## Abbreviations

The following abbreviations are used in this manuscript:

BA	Blood ammonia
HE	Hepatic encephalopathy
POC	Point-of-care
BCS	Body condition score
